# Towards Noise Simulation in Interacting Nonequilibrium Systems Strongly Coupled to Baths

**DOI:** 10.1038/s41598-017-09060-0

**Published:** 2017-08-29

**Authors:** Kuniyuki Miwa, Feng Chen, Michael Galperin

**Affiliations:** 1 Surface and Interface Science Laboratory, RIKEN, Wako, Saitama 351-0198 Japan; 2 Department of Physics, University of California San Diego, La Jolla, CA 92093 USA; 3 Department of Chemistry & Biochemistry, University of California San Diego, La Jolla, CA 92093 USA

## Abstract

Progress in experimental techniques at nanoscale makes measurements of noise in molecular junctions possible. These data are important source of information not accessible through average flux measurements. The emergence of optoelectronics, the recently shown possibility of strong light-matter couplings, and developments in the field of quantum thermodynamics are making measurements of transport statistics even more important. Theoretical methods for noise evaluation in first principles simulations can be roughly divided into approaches for weak intra-system interactions, and those treating strong interactions for systems weakly coupled to baths. We argue that due to structure of its diagrammatic expansion, and the use of many-body states as a basis of its formulation, the recently introduced nonequilibrium diagrammatic technique for Hubbard Green functions is a relatively inexpensive method suitable for evaluation of noise characteristics in first principles simulations over a wide range of parameters. We illustrate viability of the approach by simulations of noise and noise spectrum within generic models for non-, weakly and strongly interacting systems. Results of the simulations are compared to exact data (where available) and to simulations performed within approaches best suited for each of the three parameter regimes.

## Introduction

Progress in experimental techniques at nanoscale resulted in the possibility to obtain information on transport characteristics of nanojunctions beyond average flux measurements^1^. In particular, shot noise measurements in single molecule junctions were recently reported in the literature^2^. Shot noise (second cumulant in the full counting statistics of quantized charge transport) yields information not accessible through average flux measurements; it is also more sensitive to intra-molecular interactions, such as intra-system Coulomb repulsion^3^, magnetism^4–6^, and intra-molecular electron-vibration interactions^7, 8^. Probing current-carrying molecular junctions by optical means, a new field of research coined the term molecular optoelectronics^9, 10^, was introduced as another way to probe shot noise and charge fluctuations in nanojunctions^11–13^. Corresponding theory of light emission from quantum noise was recently formulated^14^. Finally, recent experiments on strong^15–17^ and ultra-strong^18^ light-molecules coupling in nano cavities makes prospects of similar measurements in molecular junctions feasible. Full counting statistics (FCS) of both charge transport and photon flux (as well as their cross-correlations) is especially important in this case.

The theoretical concept of full counting statistics was originally proposed by Levitov and Lesovik^19, 20^, and further developed in numerous studies^1^. In particular, within the standard nonequilibrium Green function (NEGF) formulation, the concept was studied by Gogolin and Komnik^21^. For noninteracting systems, the formulation is exact, and both steady-state^21–23^ and transient regime^24, 25^ considerations are available in the literature. Accounting for intra-system interactions is a nontrivial task. Currently, the two main approaches are: perturbation theory treatments for weak (relative to system-baths coupling) interactions^26–34^ and quantum master (or even rate) equations (QME) for strong interactions (and negligible system-baths coupling)^35–37^. In the former and within the NEGF, self-consistent FCS formulations^38, 39^ (as required for an approximation to be conserving^40, 41^) were considered, and first *ab initio* simulations were performed^42^. The QME approach found numerous applications in the developing field of quantum thermodynamics^43^. Both approaches have their limitations: the former is formulated in the language of elementary excitations and thus is inconvenient in treating strong intra-system interactions. The QME formulated in the basis of system many-body states (thus accounting for intra-system interactions) has difficulty treating strong system-bath couplings. In this respect, an important extension of the latter approach is formulation of the real time perturbation theory (and similar approaches)^44–50^, which allowed evaluation of shot noise accounting for system-bath coupling within a systematic perturbation expansion^51^.

In this study, we use the recently proposed nonequilibrium diagrammatic technique for Hubbard Green functions (Hubbard NEGF)^52^ to simulate FCS and noise spectrum in molecular junctions. We note in passing that earlier studies of Hubbard NEGF^53, 54^ were restricted to the evaluation of two-time correlation functions only, while simulation of noise spectrum requires evaluation of multi-time correlation functions. The latter is only possible within diagrammatic consideration (for a detailed comparison between earlier studies and diagrammatic approach to Hubbard NEGF, see ref. 52). Contrary to the standard NEGF, which considers multi-time correlation functions of elementary (de-)excitation operators ${\hat{<mml:mpadded xmlns:xlink="http://www.w3.org/1999/xlink" lspace="-1.5pt">d</mml:mpadded>}}_{\sigma }^{\dagger }$ (${\hat{d}}_{i}$) - where *i* is a single electron orbital, the Hubbard NEGF deals with correlation functions of projection operators ${\hat{X}}_{{S}_{1}{S}_{2}}=|{S}_{1}\rangle \langle {S}_{2}|$ - where |*S*_1,2_〉 are many-body states of the system. Similarities between the two approaches (illustrated by spectral decomposition ${\hat{d}}_{i}={\sum }_{{S}_{1},{S}_{2}}\langle {S}_{1}|{\hat{d}}_{i}|{S}_{2}\rangle {\hat{X}}_{{S}_{1}{S}_{2}}$) indicate that the structure of the diagrammatic technique for the Hubbard NEGF should (to some extent) resemble that of the standard NEGF (although expansions themselves are different: Hubbard NEGF considers perturbation series in system-bath coupling, while NEGF expands in small intra-system interactions). A similar way of treating self-energies in the two techniques makes expectation that Hubbard NEGF will be successful in accounting for relatively strong system-bath couplings plausible. At the same time, formulation in the language of many-body states makes the Hubbard NEGF similar to the QME approaches. Thus one may expect that accounting for strong intra-system interactions is feasible within the Hubbard NEGF. Note that similar to the real time perturbation theory of refs 44–46, 51 the Hubbard NEGF expansion is systematic. Note also that lowest order Hubbard NEGF diagrams are still simple enough to be applicable in first principles simulations. This paves a way to *ab initio* calculations of noise beyond the weak interaction limit of ref. 42.

Below, we present noise simulation in model systems within the Hubbard NEGF, comparing results to those of other approaches. The presentation is followed by a discussion of the approach indicating its strengths and weaknesses, and pointing to directions for further research. Finally, we briefly introduce the Hubbard NEGF diagrammatic technique; details of the technique and its application to noise simulation can be found in ref. 52 and in the Supplementary Information.

## Results

We consider Anderson impurity as a junction model. Its Hamiltonian is

1 $$\begin{array}{rcl}\hat{H} & = & \sum _{\sigma =\uparrow ,\downarrow }{\varepsilon }_{\sigma }{\hat{n}}_{\sigma }+U{\hat{n}}_{\uparrow }{\hat{n}}_{\downarrow }+\sum _{\begin{array}{c}k\in L,R\\ \sigma =\uparrow ,\downarrow \end{array}}{\varepsilon }_{k\sigma }{\hat{n}}_{k\sigma }+\sum _{\begin{array}{c}k\in L,R\\ {\sigma }_{1},{\sigma }_{2}=\uparrow ,\downarrow \end{array}}({V}_{{\sigma }_{1},k{\sigma }_{2}}{\hat{d}}_{{\sigma }_{1}}^{\dagger }{\hat{c}}_{k{\sigma }_{2}}+H.c.)\\  & \equiv  & \sum _{S}{E}_{S}{\hat{X}}_{SS}+\sum _{\begin{array}{c}k\in L,R;m\\ \sigma =\uparrow ,\downarrow \end{array}}{\varepsilon }_{k\sigma }{\hat{n}}_{k\sigma }+\sum _{\begin{array}{c}k\in L,R;m\\ {\sigma }_{1},{\sigma }_{2}=\uparrow ,\downarrow \end{array}}({V}_{m({\sigma }_{1}),k{\sigma }_{2}}{\hat{X}}_{m}^{\dagger }{\hat{c}}_{k{\sigma }_{2}}+H.c.)\end{array}$$

The first line utilizes second quantization, while the second employs many-body states. Here, ${\hat{<mml:mpadded xmlns:xlink="http://www.w3.org/1999/xlink" lspace="-1.5pt">d</mml:mpadded>}}_{\sigma }^{\dagger }$ and ${\hat{c}}_{k\sigma }^{\dagger }$ are creation operators for electron with spin *σ* in the system or contact state *k*, respectively; ${\hat{n}}_{\sigma }={\hat{d}}_{\sigma }^{\dagger }{\hat{d}}_{\sigma }$ and ${\hat{n}}_{k\sigma }={\hat{c}}_{k\sigma }^{\dagger }{\hat{c}}_{k\sigma }$, |*S*〉 = |0〉, |*a*〉, |*b*〉, |2〉 are empty, spin-up, spin-down, and double-occupied many-body states with energies *E*_0_ = 0, *E*_*a*_ = *ε*_↑_, *E*_*b*_ = *ε*_↓_, *E*_2_ = *ε*_↑_ + *ε*_↓_ + *U*; *m* = 0*a*, *b*2, 0*b*, *a*2 are single electron transitions between pairs of many-body states (0*a* and *b*2 are spin up transitions, 0*b* and *a*2 are spin down), and *L* and *R* are left and right contacts. Within the model, *U* represents intra-system interaction, system-baths couplings are characterized by escape rate matrices, ${{\rm{\Gamma }}}_{{\sigma }_{1}{\sigma }_{2}}^{K}(E)=2\pi {\sum }_{k\in K;\sigma }{V}_{{\sigma }_{1},k\sigma }{V}_{k\sigma ,{\sigma }_{2}}\delta (E-{\varepsilon }_{k\sigma })$ (*K* = *L*, *R*), which are energy-independent in the wide band approximation.

We simulate the current and noise spectrum at the left interface for a steady-state transport situation

2 $$\begin{array}{rcl}{I}_{L} & = & \langle {\hat{I}}_{L}(0)\rangle \quad {S}_{LL}(\omega )=\frac{1}{2}{\int }_{-\infty }^{+\infty }dt\,{e}^{i\omega t}\langle \{\delta {\hat{I}}_{L}(t);\delta {\hat{I}}_{L}(0)\}\rangle \\ {\hat{I}}_{L}(t) & = & -\frac{d}{dt}\sum _{k\in L;\sigma }{\hat{n}}_{k\sigma }(t)\quad \delta {\hat{I}}_{L}(t)={\hat{I}}_{L}(t)-\langle {\hat{I}}_{L}(t)\rangle \end{array}$$

**Figure 1:**
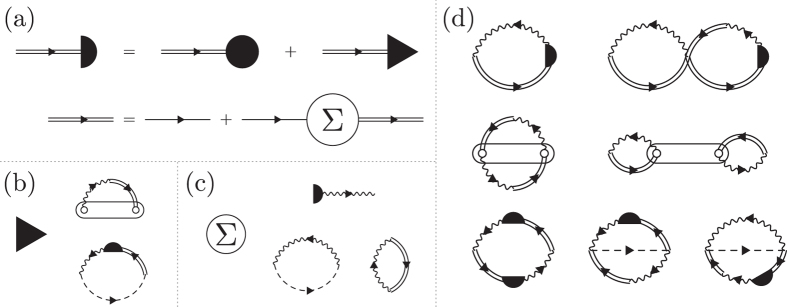
Hubbard NEGF. Shown are (**a**) generalized Dyson equation and second order dressed diagrams for (**b**) vertex Δ (triangle) and (**c**) self-energy Σ. Panel (**d**) presents diagrams utilized in noise spectrum simulations. Directed single (double) solid line represents zero-order (dressed) locator *g* ^(0)^ (*g*), directed dashed line stands for two-electron Green function, and directed wavy line indicates electron-electron correlation function yielding influence of contacts (in standard NEGF this function is called self-energy due to coupling to contacts). Circle represents spectral weight *F*, semi-circle stands for the strength operator *P* = *F* + Δ, and oval is intra-molecular correlation function. See Methods and Supporting Information for details.

Green function techniques use exact (Meir-Wingreen) expression for the current, and approximate (perturbative in system-baths couplings) derivation similar to that of ref. 30 for the noise spectrum. In the Hubbard NEGF, this derivation leads to diagrams presented in Fig. 1d. FCS is introduced as usual by dressing transfer matrix elements ${V}_{{\sigma }_{1},k{\sigma }_{2}}$ (${V}_{m({\sigma }_{1}),k{\sigma }_{2}}$) with a contour-branch-dependent counting field *λ* for *k* ∈ *L*. Zero frequency noise (second cumulant of the FCS) is obtained from a derivative of the dressed current, ${S}_{LL}(\omega =\mathrm{0)}=-i{\partial }_{\lambda }{I}_{L}^{\lambda }{|}_{\lambda =0}$ (see, e.g., ref. 55 for details). Simulations are performed within the Hubbard NEGF, standard NEGF, and Lindblad/Redfield QME for non-interacting (*U* = 0), weakly interacting (*U* ≤ Γ_0_), and strongly interacting ($U\gg {{\rm{\Gamma }}}_{0}$) cases (system-bath coupling strength Γ_0_ is used as a unit of energy). Note that Lindblad/Redfield QME does not yield noise spectrum; current and zero-frequency noise were simulated within the FCS (see ref. 56 for details). Results of the simulations are presented in units of *I*_0_ = *e*Γ_0_/${\hbar}$ for current and *S*_0_ = *e* ^2^Γ_0_/2${\hbar}$ for noise.

### Non-interacting system

We first present results of simulations for non-interacting case (*U* = 0). Note that all the standard NEGF results (including expression for noise spectrum) are exact in this case. We consider two sets of parameters representing degenerate and non-degenerate two-level systems. Parameters for the degenerate model are (all energies are in units of Γ_0_) *ε*_↑_ = *ε*_↓_ = 5, ${{\rm{\Gamma }}}_{\uparrow \uparrow }^{K}={{\rm{\Gamma }}}_{\downarrow \downarrow }^{K}=1$ and ${{\rm{\Gamma }}}_{\uparrow \downarrow }^{K}={{\rm{\Gamma }}}_{\downarrow \uparrow }^{K}=0.5$ (*K* = *L*, *R*). Non-degenerate model is given by *ε*_↑_ =−5, *ε*_↓_ = 5, ${{\rm{\Gamma }}}_{\uparrow \uparrow }^{K}={{\rm{\Gamma }}}_{\downarrow \downarrow }^{K}=1$ and ${{\rm{\Gamma }}}_{\uparrow \downarrow }^{K}={{\rm{\Gamma }}}_{\downarrow \uparrow }^{K}=0$. In both models, temperature was taken *k*_*B*_ *T* = 1/3, Fermi energy chosen as the origin (*E*_*F*_ = 0), and bias applied symmetrically (*μ*_*L*,*R*_ = *E*_*F*_ ± |*e*|*V*_*sd*_/2). Simulations were performed on an energy grid spanning from −120 to 120 with step 0.01. Tolerance for convergence of the Hubbard NEGF scheme was 0.001 for the difference in density matrix values (state populations and coherences) in consequent steps of iteration. The counting field for numerical evaluation of FCS was chosen as *λ* = 0.01.

**Figure 2:**
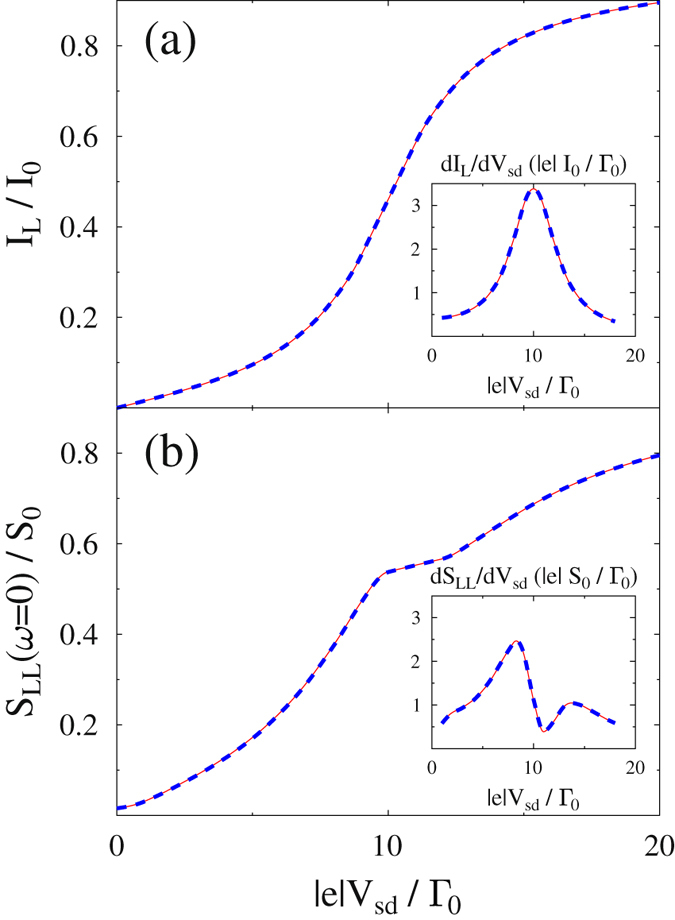
Degenerate non-interacting two-level system. Shown are (**a**) current and (**b**) zero frequency noise obtained employing full counting statistics within the Hubbard NEGF (dashed line, blue) and standard NEGF (solid line, red) approaches. For non-interacting case the latter is exact. Insets show conductance and differential noise, respectively. See text for parameters.

**Figure 3:**
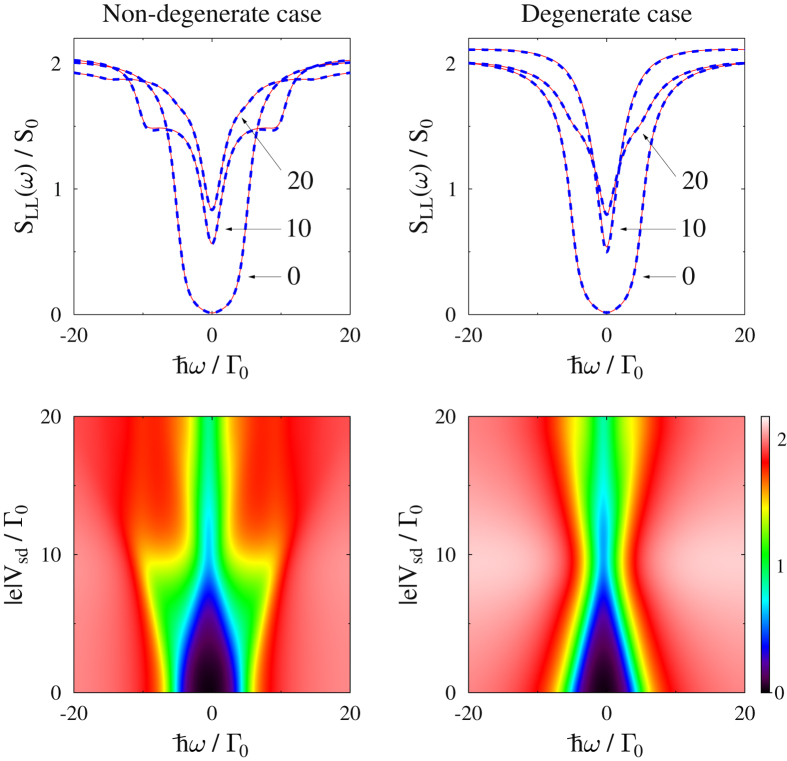
Non-degenerate (left) and degenerate (right) non-interacting two-level systems. Shown is noise spectrum simulated within the Hubbard NEGF utilizing diagrams in Fig. 1d. Top panels (horizontal cuts of lower maps) compare the Hubbard NEGF (dashed line, blue) with exact results (solid line, red) for three biases. See text for parameters.

Figure 2 presents results of FCS simulations for the degenerate model, performed within the Hubbard NEGF (dashed line) and standard NEGF (solid line). The Hubbard NEGF employs self-consistent FCS as described in ref. 38. FCS results were checked against analytical expressions known for noninteracting systems. Standard NEGF (exact) results are reproduced very accurately by the approximate Hubbard NEGF simulations for both current (Fig. 2a) and zero-frequency noise (Fig. 2b). At resonant bias, |*e*|*V*_*sd*_ = 10Γ_0_, conductance is maximum (inset in Fig. 2a), while noise derivative has a dip (inset in Fig. 2b), as is expected from the Landauer expression for shot noise. Results for the non-degenerate model are similar. The noise spectrum simulated for the two models within the Hubbard NEGF also reproduces exact (NEGF) results accurately (see Fig. 3).

Note that pseudoparticle NEGF (PP-NEGF), another popular Green function methodology using system many-body states, is widely employed as a standard impurity solver in nonequilibrium dynamical mean field theory simulations^57^. The inability of the methodology to yield information on full counting statistics presumably comes from its formulation within extended (unphysical) Hilbert space. At the same time, PP-NEGF can be used for simulations of current and noise spectrum. In Supplementary Information, we show that at the same (second order) level of diagrammatic perturbation expansion in system-baths couplings, PP-NEGF reproduces current, but fails to reproduce noise spectrum. The method is unable to yield correct spectral function even for non-interacting systems, which leads to failure of the noise spectrum simulation.

### Weakly interacting system

**Figure 4:**
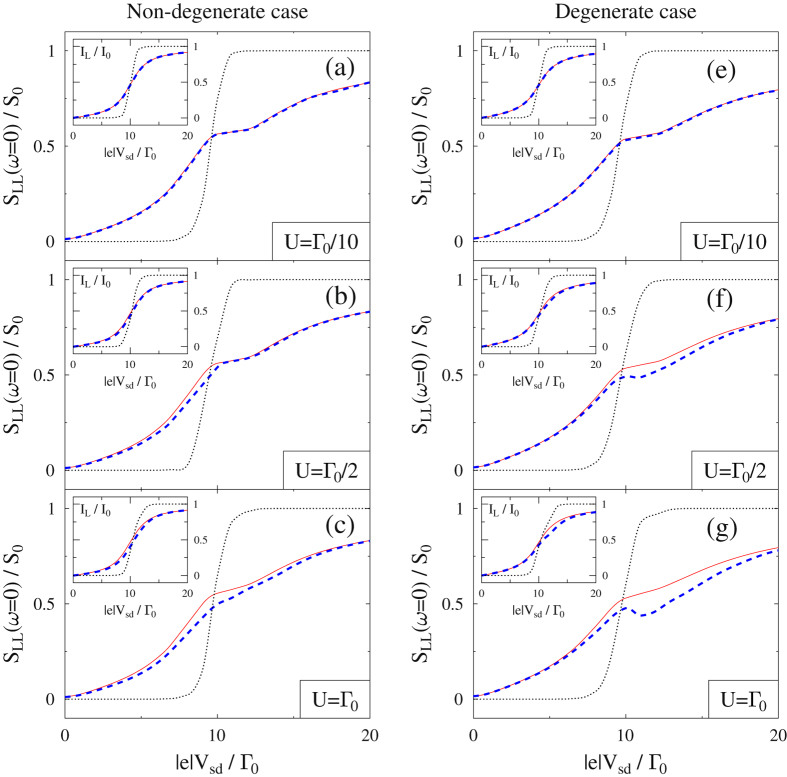
Non-degenerate (**a**–**c**) and degenerate (**e**–**g**) Hubbard model in the weak interaction *U* ≤ Γ_0_ regime. Shown are current (insets) and zero-frequency noise vs. applied bias. Simulations are performed utilizing the full counting statistics within the Hubbard NEGF (dashed line, blue), standard NEGF (solid line, red), and Lindblad/Redfield QME (dotted line, black). See text for parameters.

We now turn to weakly interacting cases, where $U/{{\rm{\Gamma }}}_{0}\ll 1$ and perturbative expansion in this small parameter within standard NEGF should yield reasonable results^58^, while Lindblad/Redfield QME is not expected to be accurate. Figure 4 shows the results of FCS simulations for both models performed within the Hubbard NEGF (dashed line), standard NEGF (solid line), and Lindblad/Redfield QME (dotted line) for a set of intra-system interaction strengths *U*. Here both Green function techniques rely on self-consistency in numerical evaluation of FCS. As expected, results are quite similar for both Green function techniques and differ significantly from the QME calculations. Naturally, Hubbard and standard NEGF results coinciding at weak *U* (see Fig. 4a and e) start to depart from each other at stronger interaction values. The difference is more pronounced in zero-frequency noise (main panels) than in the current (insets). There is an interesting distinction between noise results for the two models: Comparing Fig. 4c and g shows that for *U* = Γ_0_, the Hubbard NEGF noise result for degenerate model develops a dip at $|e|{V}_{sd} \sim 10{{\rm{\Gamma }}}_{0}$, while no dip is observed in the non-degenerate case. The reason is difference in energetics of the two models: Single electron transitions 0*a*, *b*2, 0*b*, *a*2 for the non-degenerate model occur at −5, −4, 5, 6, while for the degenerate model the transitions are at 5, 6, 5, 6 (all energies are in units of Γ_0_). Thus by the time bias reaches a resonant value of |*e*|*V*_*sd*_ = 10Γ_0_ (when physical electronic population of the system becomes significant) channel *b*2 in the non-degenerate model is open so that state |2〉 can be populated. Therefore no Coulomb blockade is observed. The degenerate model is Coulomb blockaded until |*e*|*V*_*sd*_ = 12Γ_0_. Such suppression of shot noise due to charging effects was observed experimentally^3^. Note that the effect is seen already at *U* = Γ_0_/2 (compare Fig. 4b and f), where perturbation expansion in *U*/Γ_0_ should still be relatively accurate. However standard NEGF does not reproduce the effect because of a mean field character of treatment of *U* within second-order diagrammatic expansion. At the same time, the Hubbard NEGF (also considered at the second order) is accurate enough to account for the suppression.

### Strongly interacting system

**Figure 5:**
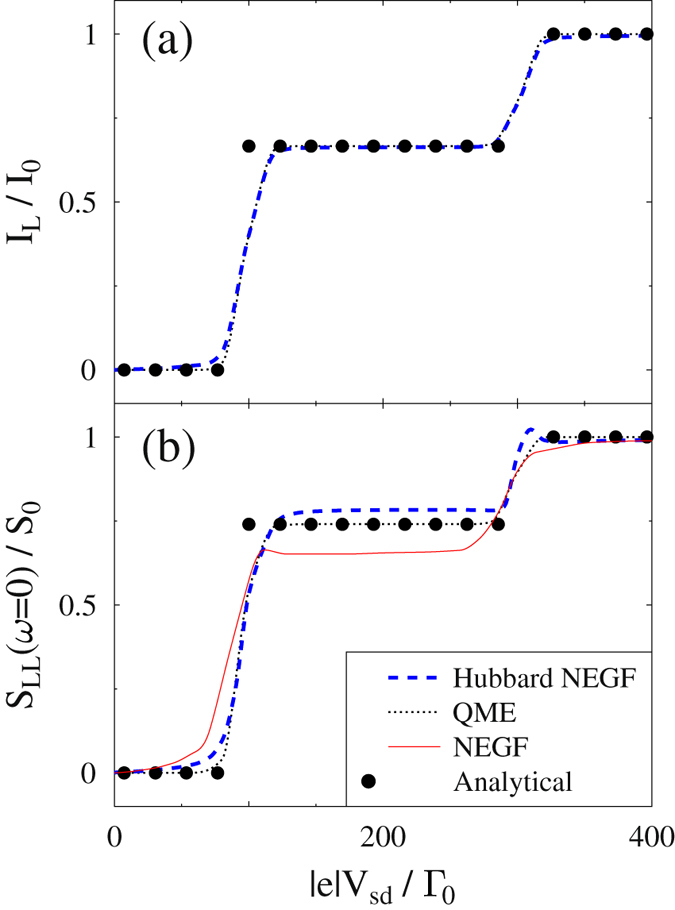
Hubbard model in the strong interaction regime $U\gg {{\rm{\Gamma }}}_{0}$. Shown are (**a**) current and (**b**) zero-frequency noise vs. applied bias. calculated within the Hubbard NEGF (dashed line, blue), standard NEGF (solid line, red), and Lindblad/Redfield QME (dotted line, black). Solid circles (black) indicate analytical result as derived in ref. 51 within the lowest (first-) order perturbation theory in Γ_0_. Noise simulations are performed within the Hubbard NEGF and standard NEGF utilizing noise spectrum diagrams of Fig. 1d and ref. 30, respectively; simulations within Lindblad/Redfield QME utilize full counting statistics. See text for parameters.

Finally, we consider strong interaction cases, $U/{{\rm{\Gamma }}}_{0}\gg 1$, where Lindblad/Redfield is expected to be relatively accurate. Following ref. 30 we consider a model with the following parameters, in which all energies are in units of Γ_0_: *ε*_↑_ = *ε*_↓_ = 50, *U* = 100, ${{\rm{\Gamma }}}_{\uparrow \uparrow }^{K}={{\rm{\Gamma }}}_{\downarrow \downarrow }^{K}=1$ and ${{\rm{\Gamma }}}_{\uparrow \downarrow }^{K}={{\rm{\Gamma }}}_{\downarrow \uparrow }^{K}=0$ (*K* = *L*, *R*), *k*_*B*_ *T* = 3. Figure 5 (analog of Fig. 7 in ref. 30) shows current and zero frequency noise simulated within the Hubbard NEGF (dashed line), standard NEGF (solid line), and Lindblad/Redfield QME (dotted line). Also shown are analytical results of ref. 51. Note that the standard NEGF consideration here follows ref. 30. Hubbard NEGF follows quite closely the expected correct behavior (QME and analytical results).

We note that diagrammatic Hubbard NEGF is a perturbative (in system-bath coupling) method, and as such is not capable to treat strong system-bath correlations. In particular, the Kondo regime is beyond the capabilities of the method. At the same time, there are many cases (including most experimental measurements of noise in molecular junctions) where strong system-bath coupling is accompanied by non-negligible intra-system interactions (e.g. electron-vibration coupling in the molecule), and yet is outside of the Kondo regime. Theoretical simulations of noise in such systems are complicated within both standard NEGF and QME approaches. The Hubbard NEGF methodology presented yields a practical tool for first principle simulations in such systems.

## Discussion

Results of simulations show that the Hubbard NEGF is an inexpensive method capable to reproduce satisfactory noise characteristics of junctions over a wide range of parameters. Indeed, one has to go only to lowest (second) order in perturbative expansion in system-bath coupling to get reliable results. We think that this universality is due to basic components of the Hubbard NEGF formulation. First, as a methodology using many-body states of the system, the Hubbard NEGF accounts for strong intra-system interactions. This also makes it similar to QME considerations, which even at the lowest (second) order in system-bath coupling are successfully utilized for FCS simulations in interacting systems weakly coupled to their surroundings. In particular, such QME methods are the cornerstone of quantum thermodynamics and nonlinear optical spectroscopy considerations. At the same time, similar to the standard NEGF and contrary to both QME and PP-NEGF formulations, the Hubbard NEGF considers time correlations between single electron transitions. Note that the standard NEGF yields exact FCS results for non-interacting systems, and was successfully applied in first principles simulations in the regime of strong coupling to baths and weak intra-system interactions. These similarities with different techniques, each most suitable at opposite limits, make the Hubbard NEGF a good candidate for a relatively inexpensive approach capable of predicting noise properties in transport junctions over a wide range of parameters.

It is interesting to note that in terms of structure of diagrammatic expansion, PP-NEGF, which considers correlation functions of many-body states, is closer to QME considerations. Thus, failure of the approach to predict zero-frequency noise in non-interacting systems at the same (second) order of perturbation theory where Hubbard NEGF was successful is expected. Besides, the very formulation of the PP-NEGF in extended (unphysical) Hilbert space presumably does not allow FCS formulation within the method. The latter was shown to work well in the case of Hubbard NEGF.

We show that the Hubbard NEGF yields accurate results over a broad range of parameters already at the lowest (second) order in the system-bath coupling. For non-interacting cases, Hubbard NEGF yields results similar to the standard NEGF, which is exact in this limit. For weak intra-system interactions, Hubbard NEGF is better than both the (same order of perturbation theory) standard NEGF and QME approaches. At the same time, for strong intra-system interactions, real-time perturbation theory works better than the present Hubbard NEGF consideration. Thus, careful (diagram to diagram) comparison of the two approaches is one of goals for future studies.

With respect to relevance of the methodology to experiments, we note that almost all noise measurements at molecular junctions are restricted to either non-interacting or relatively weak electron-vibration interactions^2, 7, 8, 59^ and are treated theoretically within second-order perturbation theory^31, 33, 34, 42^. Hubbard NEGF is a scheme applicable in such experimentally relevant situations, and is capable of going beyond lowest-order perturbative schemes in intra-system interactions. It also may serve as a useful theoretical tool for investigation of (soon expected to be reported) measurements of strong light-matter interactions^15–18^ in molecular junctions.

## Methods

Details on standard NEGF simulations can be found in refs 30, 38. Lindblad/Redfield simulations are discussed in refs 43, 56. PP-NEGF formulation is discussed in refs 57, 60. Expressions for noise spectrum within the PP-NEGF can be found in Supporting Information. Here we briefly discuss the Hubbard NEGF approach.

Hubbard NEGF considers the correlation function of Hubbard operators defined on the Keldysh contour as

3 $${G}_{{m}_{1}{m}_{2}}({\tau }_{1},{\tau }_{2})=-i\langle {T}_{c}\,{\hat{X}}_{{m}_{1}}({\tau }_{1})\,{\hat{X}}_{{m}_{2}}^{\dagger }({\tau }_{2})\rangle $$

where *τ*_1,2_ are contour variables, *T*_*c*_ is contour ordering operator, *m*_1,2_ are single electron transitions between many body states, and ${\hat{X}}_{m}=|{S}_{1}\rangle \langle {S}_{2}|$ with |*S*_1_〉 containing one electron less than |*S*_2_〉. The Green function is obtained by solving a modified version of the Dyson type equation (see Fig. 1a)

4 $$\begin{array}{rcl}{G}_{{m}_{1}{m}_{2}}({\tau }_{1},{\tau }_{2}) & = & \sum _{{m}_{3}}{\int }_{c}d{\tau }_{3}\,{g}_{{m}_{1}{m}_{3}}({\tau }_{1},{\tau }_{3}){P}_{{m}_{3}{m}_{2}}({\tau }_{3},{\tau }_{2})\\ {g}_{{m}_{1}{m}_{2}}({\tau }_{1},{\tau }_{2}) & = & {g}_{{m}_{1}{m}_{2}}^{0}({\tau }_{1},{\tau }_{2})+\sum _{{m}_{3},{m}_{4}}{\int }_{c}d{\tau }_{3}{\int }_{c}d{\tau }_{4}{g}_{{m}_{1}{m}_{3}}^{0}({\tau }_{1},{\tau }_{3}){{\rm{\Sigma }}}_{{m}_{3}{m}_{4}}({\tau }_{3},{\tau }_{4}){g}_{{m}_{4}{m}_{2}}({\tau }_{4},{\tau }_{2})\end{array}$$

Here, ${g}_{{m}_{1}{m}_{2}}({\tau }_{1},{\tau }_{2})$ is the locator, ${g}_{{m}_{1}{m}_{2}}^{0}({\tau }_{1},{\tau }_{2})$ is the locator in the absence of coupling to the baths, ${{\rm{\Sigma }}}_{{m}_{1}{m}_{2}}({\tau }_{1},{\tau }_{2})$ is self-energy, and ${P}_{{m}_{1}{m}_{2}}({\tau }_{1},{\tau }_{2})$ is the strength operator. The latter is the sum of spectral weight ${F}_{{m}_{1}{m}_{2}}(\tau )$ and vertex ${{\rm{\Delta }}}_{{m}_{1}{m}_{2}}({\tau }_{1},{\tau }_{2})$ functions (dressed second-order diagrams for vertex Δ and self-energy Σ are shown in Fig. 1b and c, respectively). Self-consistency of the approach comes from the fact that both self-energy and strength operators depend on the Hubbard Green function. After convergence, the Hubbard Green function is used in a simulation of current and noise. For FCS, the Eq (4) are dressed with counting fields. Details of the self-consistent procedure are given in ref. 52. Explicit expressions for noise spectrum in terms of Hubbard Green functions are given in the Supporting Information. Figure 1d shows corresponding diagrams.

### Data Availability Statement

All data generated or analyzed during this study are included in this published article (and its Supplementary Information files).

## Electronic supplementary material

Supporting Information: Towards Noise Simulation in Interacting Nonequilibrium Systems Strongly Coupled to Baths
