# Supplementary material for: Towards Noise Simulation in Interacting Nonequilibrium Systems Strongly Coupled to Baths
Source: Sci Rep. 2017 Aug 29;7:9735. doi: 10.1038/s41598-017-09060-0 (PMC5574948; doi:10.1038/s41598-017-09060-0)
Supplement: Supplementary file 1 — Supporting Information: Towards Noise Simulation in Interacting Nonequilibrium Systems Strongly Coupled to Baths [file 41598_2017_9060_MOESM1_ESM.pdf]

Supporting Information: Towards Noise Simulation in Interacting Nonequilibrium Systems Strongly Coupled to Baths

Kuniyuki Miwa[1], Feng Chen[2], and Michael Galperin[3,*]

[1]Surface and Interface Science Laboratory, RIKEN, Wako, Saitama 351-0198, Japan
[2]Department of Physics, University of California San Diego, La Jolla, CA 92093, USA
[3]Department of Chemistry & Biochemistry, University of California San Diego, La Jolla, CA 92093, USA
[*]migalperin@ucsd.edu

ABSTRACT

Here we present supporting information and technical details of simulations of current and noise within the Hubbard and pseudoparticle NEGF.

Hubbard NEGF

Here we present details of the Hubbard NEGF simulation.

Equation of motion

Equation of motion for the Hubbard NEGF (the generalized Dyson equation) is Eq.(4) of the paper. Its diagrammatic representation is given in Fig. 1a of the paper. Dynamics of the Hubbard Green function (3) is expressed in terms of locator g, self-energy due to coupling to contacts Σ, spectral weight F, vertex Δ, strength operator $P \equiv F + \Delta$, correlation function C, and standard NEGF self-energy σ. Difference between the Hubbard NEGF G and locator is the strength operator P (see top row in Eq.(4) of the paper). This is direct consequence of Hubbard operators commutation relations, $[\hat{X}_{S_1S_2};\hat{X}^\dagger_{S_3S_4}]_\pm = \delta_{S_2,S_4}\hat{X}_{S_1S_3} \pm \delta_{S_1,S_3}\hat{X}_{S_4S_2}$, which (contrary to the usual quasiparticle algebra) yield an operator as result of (anti)commutation. Locator g satisfies equation of motion structurally similar to the usual Dyson equation (see bottom row in Eq.(4) of the paper). Eq.(4) of the paper is exact in the same sense as the usual Dyson equation. It is represented on the Keldysh contour and have to be solved self-consistently because the self-energy Σ and strength operator P depend on the Hubbard Green function G, which in turn is obtained from them. Our calculations were performed at the second order (in system-baths coupling) of the Hubbard NEGF diagrammatic perturbation theory. Explicit expressions for Σ, F, Δ, P, and C can be found in Ref.[1]. Diagrams for Δ and Σ are shown in Figs. 1b and 1c of the paper, respectively.

Full counting statistics is introduced as usual dressing the Keldysh contour with counting fields (see, e.g., Ref.[2] for details). In particular, electron electron counting statistics at interface K (L or R) is obtained by dressing the standard NEGF self-energy σ^K as follows

$$\sigma^K(t_1,t_2) = \begin{bmatrix} \sigma^{K\,00}(t_1,t_2) & -\sigma^{K\,01}(t_1,t_2)\,e^{i\lambda} \\ -\sigma^{K\,10}(t_1,t_2)\,e^{-i\lambda} & \sigma^{K\,11}(t_1,t_2) \end{bmatrix} \tag{1}$$

Here matrix 2×2 yields contour representation of the self-energy; each block is 4×4 matrix in single-electron transitions between many-body states of the molecule. Thus dressed σ^K is utilized in EOM (4) of the paper. After the EOM converges, dressed current at steady-state is obtained as usual

$$I_K^\lambda = \frac{e}{\hbar}\int\frac{dE}{2\pi}\mathrm{Tr}\left[\sigma^{K\,01}(E)\,e^{i\lambda}\,G^{10}(E) - \sigma^{K\,10}(E)\,e^{-i\lambda}\,G^{01}(E)\right] \tag{2}$$

where $\mathrm{Tr}[\ldots]$ is over single-electron transitions between many-body states of the molecule. Setting $\lambda = 0$ yields usual expression for current at interface K, while taking (numerically) derivative in the counting field λ yields zero-frequency noise $S_KK(\omega=0) = -i\partial_\lambda I_K^\lambda|_{\lambda=0}$. We note that in the strongly interacting regime we had difficulty converging the EOM (4) when σ is dressed (i.e. for non-zero λ). In this regime zero-frequency noise was calculated from the noise spectrum expression (see below).

Noise spectrum

Derivation of an approximate expression for noise spectrum starts from the definition (2) and proceeds with second order perturbation theory consideration in molecule-contacts coupling (last term in Eq.(1) of the paper). As a result on has to evaluate products of multi-time correlation functions of contact operators $\hat{c}_{k\sigma}$ and Hubbard operators $\hat{X}_m$. The former are evaluated utilizing the standard Wick's theorem, while the latter follow the Hubbard NEGF diagrammatic technique as introduced in Ref.[1]. This leads to the following expression for noise spectrum (here and below $e=\hbar=1$)

$$S_{K_1K_2}(t_1,t_2)=\sum_{i=1}^{7} S^{(i)}_{K_1K_2}(t_1,t_2) \tag{3}$$

where $K_{1,2}=L,R$ and

$$S^{(1)}_{K_1K_2}(t_1,t_2)=\delta_{K_1,K_2}\frac{1}{2}\sum_{m_1,m_2}\sum_{s=0,1}\left(\sigma^{K_1\,s(1-s)}_{m_1m_2}(t_1,t_2)G^{(1-s)s}_{m_2m_1}(t_2,t_1)+(1\leftrightarrow 2)\right) \tag{4}$$

$$\begin{aligned}S^{(2)}_{K_1K_2}(t_1,t_2)=&\frac{1}{2}\sum_{\{m_i\}}\sum_{\{s_i=0,1\}}\int dt_3\int dt_4\,\tau^z_{s_3s_3}\tau^z_{s_4s_4}\Big(\\
&g^{1s_3}_{m_1m_2}(t_1,t_3)\sigma^{K_1\,s_3 1}_{m_3m_1}C^{s_3s_4}_{m_2m_3,m_4m_5}(t_3,t_4)g^{0s_4}_{m_6m_4}(t_2,t_4)\sigma^{K_2\,s_4 0}_{m_5m_6}(t_4,t_2)\\
&+\Sigma^{K_1\,0s_3}_{m_1m_3}(t_1,t_3)g^{s_3 1}_{m_3,m_5}(t_3,t_1)\Sigma^{K_2\,0s_4}_{m_2m_4}(t_2,t_4)g^{s_3 1}_{m_4,m_6}(t_4,t_2)C^{10}_{m_5m_1,m_6m_2}(t_1,t_2)\\
&-\sigma^{K_2\,0s_4}_{m_2m_4}(t_2,t_3)g^{s_3 1}_{m_4,m_6}(t_3,t_2)\sum_{s=0}^{1}g^{ss_3}_{m_1m_3}(t_1,t_3)\sigma^{K_1\,s_3 s}_{m_5m_1}C^{s_3(1-s)}_{m_3m_5,m_6m_2}(t_3,t_2)+(1\leftrightarrow 2)\Big)\end{aligned} \tag{5}$$

$$\begin{aligned}S^{(3)}_{K_1K_2}(t_1,t_2)=&\frac{1}{2}\sum_{\{m_i\}}\sum_{\{s_i=0,1\}}\int dt_3\int dt_4\,\tau^z_{s_3s_3}\tau^z_{s_4s_4}\Big(\\
&-g^{1s_3}_{m_1m_3}(t_1,t_3)\sigma^{K_2\,s_3 0}_{m_5m_2}(t_3,t_2)C^{s_3s_4}_{m_3m_5,m_4m_6}(t_3,t_4)g^{0s_4}_{m_2m_4}(t_2,t_4)\sigma^{K_1\,s_4 1}_{m_6m_1}(t_4,t_1)\\
&-\sigma^{K_1\,1s_3}_{m_1m_3}(t_1,t_3)g^{s_3 0}_{m_3m_6}(t_3,t_2)\sigma^{K_2\,0s_4}_{m_2m_4}(t_2,t_4)g^{s_4 1}_{m_4m_5}(t_4,t_1)C^{10}_{m_5m_1,m_6m_2}(t_1,t_2)\\
&+\sum_{s=0,1}g^{s(1-s)}_{m_1m_4}(t_1,t_2)\sigma^{K_2\,(1-s)s_3}_{m_2m_3}(t_2,t_3)g^{s_3s_4}_{m_3m_5}(t_3,t_4)\sigma^{K_1\,s_4 s}_{m_6m_1}(t_4,t_1)C^{s_4(1-s)}_{m_5m_6,m_4m_2}+(1\leftrightarrow 2)\Big)\end{aligned} \tag{6}$$

$$\begin{aligned}S^{(4)}_{K_1K_2}(t_1,t_2)=&\frac{i}{2}\sum_{\{m_i\}}\sum_{\{s_i=0,1\}}\int dt_3\int dt_4\,\tau^z_{s_3s_3}\tau^z_{s_4s_4}\Big(\\
&\eta(m_6,m_5m_3)g^{1s_3}_{m_1m_5}(t_1,t_3)\sigma^{K_1\,s_3 1}_{m_3m_1}(t_3,t_1)G^{s_3s_4}_{\gamma(m_6,m_5m_3)m_4}(t_3,t_4)\sigma^{K_2\,s_4 0}_{m_4m_2}(t_4,t_2)g^{0s_3}_{m_2m_6}(t_2,t_3)\\
&-\eta(m_6,m_5m_3)g^{0s_3}_{m_1m_6}(t_1,t_3)\sigma^{K_1\,s_3 0}_{m_3m_1}(t_3,t_1)G^{s_3s_4}_{\gamma(m_6,m_5m_3)m_4}(t_3,t_4)\sigma^{K_2\,s_4 1}_{m_4m_2}(t_4,t_2)g^{1s_3}_{m_2m_5}(t_2,t_3)\\
&+\eta(m_6,m_5m_1)\sigma^{K_1\,1s_3}_{m_1m_3}(t_1,t_3)g^{s_3 1}_{m_3m_5}(t_3,t_1)G^{10}_{\gamma(m_6,m_5m_1)m_2}(t_1,t_2)\sigma^{K_2\,0s_4}_{m_2m_4}(t_2,t_4)g^{s_4 1}_{m_4m_6}(t_4,t_1)\\
&-\eta(m_6,m_5m_1)\sigma^{K_1\,0s_3}_{m_1m_3}(t_1,t_3)g^{s_3 0}_{m_3m_6}(t_3,t_1)G^{01}_{\gamma(m_6,m_5m_1)m_2}(t_1,t_2)\sigma^{K_2\,1s_4}_{m_2m_4}(t_2,t_4)g^{s_4 0}_{m_4m_5}(t_4,t_1)\\
&-\sum_{s=0,1}\eta(m_6,m_5m_3)g^{ss_3}_{m_1m_5}(t_1,t_3)\sigma^{K_1\,s_3 s}_{m_3m_1}(t_3,t_1)\sigma^{K_2\,(1-s)s_4}_{m_2m_4}(t_2,t_4)g^{s_4s_3}_{m_4m_6}(t_4,t_3)G^{s_3(1-s)}_{\gamma(m_6,m_5m_3}(t_3,t_2)\\
&+\sum_{s=0,1}\eta(m_6,m_5m_1)\sigma^{K_1\,ss_3}_{m_1m_3}(t_1,t_3)g^{s_3 s}_{m_3m_6}(t_3,t_1)G^{ss_4}_{\gamma(m_6,m_5m_1)m_4}(t_1,t_4)\sigma^{K_2\,s_4(1-s)}_{m_4m_2}(t_4,t_2)g^{(1-s)s}_{m_2m_5}(t_2,t_1)\\
&+(1\leftrightarrow 2)\Big)\end{aligned} \tag{7}$$

$$\begin{aligned}S^{(5)}_{K_1K_2}(t_1,t_2)=&\frac{1}{2}\sum_{\{m_i\}}\sum_{\{s_i=0,1\}}\int dt_3\int dt_4\,\tau^z_{s_3s_3}\tau^z_{s_4s_4}\Big(\\
&-G^{1s_3}_{m_1m_3}(t_1,t_3)\sigma^{K_2\,s_3 0}_{m_3m_2}(t_3,t_2)G^{0s_4}_{m_1m_4}(t_1,t_4)\sigma^{K_1\,s_4 1}_{m_4m_1}(t_4,t_1)\\
&+\sigma^{K_1\,1s_3}_{m_1m_3}(t_1,t_3)G^{s_2 0}_{m_3m_2}(t_3,t_2)\sigma^{K_2\,0s_4}_{m_2m_4}(t_2,t_4)G^{s_4 1}_{m_4m_1}(t_4,t_1)\\
&+\sum_{s=0,1}G^{s(1-s)}_{m_1m_2}(t_1,t_2)\sigma^{K_2\,(1-s)s_4}_{m_2m_4}(t_2,t_4)G^{s_4s_3}_{m_4m_3}(t_4,t_3)\sigma^{K_1\,s_3 s}_{m_3m_1}(t_3,t_1)+(1\leftrightarrow 2)\Big)\end{aligned} \tag{8}$$

$$S^{(6)}_{K_1K_2}(t_1,t_2) = \frac{i}{2}\sum_{\{m_i\}}\sum_{\{s_i=0,1\}}\int dt_3\int dt_4\,\tau^z_{s_3s_3}\tau^z_{s_4s_4}\Big(+(-1)^{m_1} d^{s_4s_1}(t_4,t_1)\sigma^{K_1\,1s_3}_{m_1m_3}(t_1,t_3)g^{s_3s_4}_{m_3\bar{m}_4}(t_3,t_4)G^{10}_{\bar{m}_1m_2}(t_1,t_2)\sigma^{K_2\,0s_4}_{m_2m_4}(t_2,t_4) +(-1)^{m_3} d^{0s_3}(t_2,t_3)G^{s_3s_4}_{\bar{m}_3m_4}(t_3,t_4)\sigma^{K_2\,s_40}_{m_4m_2}(t_4,t_2)\sigma^{K_1\,s_31}_{m_3m_1}(t_3,t_1)g^{10}_{m_1\bar{m}_2}(t_1,t_2) - \sum_{s=0,1}(-1)^{m_3} d^{s_4s_3}(t_4,t_3)\sigma^{K_1\,s_3s}_{m_3m_1}(t_3,t_1)g^{ss_4}_{m_1\bar{m}_4}(t_1,t_4)G^{s_3(1-s)}_{\bar{m}_3m_2}(t_3,t_2)\sigma^{K_2\,(1-s)s_4}_{m_2m_4}(t_2,t_4) + (1\leftrightarrow 2)\Big) \tag{9}$$

$$S^{(7)}_{K_1K_2}(t_1,t_2) = \frac{i}{2}\sum_{\{m_i\}}\sum_{\{s_i=0,1\}}\int dt_3\int dt_4\,\tau^z_{s_3s_3}\tau^z_{s_4s_4}\Big(-(-1)^{m_3} d^{0s_3}(t_1,t_3)\sigma^{K_1\,s_30}(t_3,t_1)G^{s_3s_4}_{\bar{m}_3m_4}(t_3,t_4)\sigma^{K_2\,s_41}_{m_4m_2}(t_4,t_2)g^{10}_{m_2\bar{m}_1}(t_2,t_1) -(-1)^{m_1} d^{s_30}(t_3,t_1)\sigma^{K_1\,0s_3}(t_1,t_3)G^{01}_{\bar{m}_1m_2}(t_1,t_2)\sigma^{K_2\,1s_4}_{m_2m_4}(t_2,t_4)g^{s_4s_3}_{m_4\bar{m}_3}(t_4,t_3) + \sum_{s=0,1}(-1)^{m_1} d^{s_3s}(t_3,t_1)\sigma^{K_1\,ss_3}(t_1,t_3)G^{ss_4}_{\bar{m}_1m_4}(t_1,t_4)\sigma^{K_2\,s_4(1-s)}_{m_4m_2}(t_4,t_2)g^{(1-s)s_3}_{m_2\bar{m}_3}(t_2,t_3) + (1\leftrightarrow 2)\Big) \tag{10}$$

Here τ^z is Pauli matrix, $s=0$ (1) indicates casual (anti-casual) branch of the Keldysh contour, m counts single electron transitions between meany-body states of the molecule, and $\bar{m}$ is transition opposite to m: $\bar{m}=3-m$. Corresponding diagrams are presented in Fig. 1d of the paper.

d in (4)-(10) is two-electron locator (see Ref.[1] for details). In our simulations we utilized quasiparticle limit for this Green function

$$\mathbf{d}(E) = \frac{1}{P_0-P_2}\begin{bmatrix} \frac{P_0}{E-E_{20}+i\delta} - \frac{P_2}{E-E_{20}-i\delta} & -2\pi i P_2\,\delta(E-E_{20}) \\ -2\pi i P_0\,\delta(E-E_{20}) & \frac{P_2}{E-E_{20}+i\delta} - \frac{P_0}{E-E_{20}-i\delta} \end{bmatrix} \tag{11}$$

Here P_0 and P_2 are probabilities to populate empty and double occupied states, respectively.

Pseudoparticle NEGF

Here we present details and results of the PP-NEGF simulations.

Equation of motion

Pseudoparticle (PP) NEGF is a popular impurity solver utilized within the dynamical mean field theory approach to strongly correlated materials.[3] PP-NEGF utilizes a second quantization in the space of many-body states $\{|S\rangle\}$. For example, state (pseudoparticle) S is constructed by applying creation operator $\hat{p}^\dagger_S$ to unphysical vacuum $|vac\rangle$: $|S\rangle = \hat{p}^\dagger_S|vac\rangle$. Pseudoparticle operators satisfy the usual commutation or anticommutation rules depending on the Bose (e.g., even number of electrons) or Fermi (e.g., odd number of electrons) character of the corresponding many-body state: $[\hat{p}_{S_1};\hat{p}^\dagger_{S_2}]_\pm = \delta_{S_1,S_2}$ and $[\hat{p}_{S_1};\hat{p}_{S_2}]_\pm = [\hat{p}^\dagger_{S_1};\hat{p}^\dagger_{S_2}]_\pm = 0$. The formulation is only possible in an extended Hilbert space, physical subspace of which is determined by the normalization condition $\sum_S \hat{p}^\dagger_S\hat{p}_S = 1$.

In the pseudoparticle representation Hamiltonian (1) of the paper reads

$$\hat{H} = \sum_S E_S\hat{p}^\dagger_S\hat{p}_S + \sum_{k\in L,R}\sum_{\sigma=\uparrow,\downarrow}\varepsilon_{k\sigma}\hat{n}_{k\sigma} + \sum_{k\in L,R}\sum_{\sigma_1,\sigma_2=\uparrow,\downarrow}\sum_{S_1,S_2}\left(V_{\sigma_1,k\sigma_2}\,\xi^{\sigma_1}_{S_2S_1}\hat{p}^\dagger_{S_2}\hat{p}_{S1}\hat{c}_{k\sigma_2} + H.c.\right). \tag{12}$$

Here $\xi^\sigma_{S_2S_1} = \langle S_2|\hat{d}^\dagger_\sigma|S_1\rangle$.

PP-NEGF considers correlation function of pseudoparticle operators defined on the Keldysh contour as

$$g_{S_1S_2}(\tau_1,\tau_2) = -i\langle T_c\,\hat{p}_{S_1}(\tau_1)\hat{p}^\dagger_{S_2}(\tau_2)\rangle \tag{13}$$

In the extended Hilbert space this Green function satisfies usual Dyson equation; usual diagrammatic perturbation expansion (in system-baths coupling) is employed to derive form of a self-energy. Restricting the consideration to physical subspace modifies structure of equation of motion (for details see, e.g, Refs.[3,4]).

Spectral decomposition, $\hat{d}^\dagger_\sigma = \sum_{S_1,S_2}\xi^\sigma_{S_2,S_1}\hat{p}^\dagger_{S_2}\hat{p}_{S_1}$, yields connection between standard NEGF single particle and PP-NEGF two-particle Green functions. At the lowest (second) level of diagrammatic perturbation expansion (the non-crossing

approximation - NCA - this is the level of theory employed in our considerations) the latter is expressed as a product of single particle pseudoparticle Green functions. Thus, after self-consistent PP-NEGF procedure is converged, single pseudoparticle Green functions (13) yield standard NEGF single-particle GF, which in turn may be utilized in simulations of spectral function and current. We note that PP-NEGF formulation in an extended Hilbert space presumably makes it inapplicable for FCS formulation. Below we detail procedure of noise spectrum simulation and present several results.

Noise spectrum

Approximate expression for noise spectrum is derived along the same lines as discussed above. This time we have to evaluate multi-time correlation function of pseudoparticle operators and employ PP-NBEGF diagrammatic technique. This leads to the following expression for noise spectrum at the left interface

$$S_{LL}(t_1,t_2) = \sum_{i=1}^{6} S_{LL}^{(i)}(t_1,t_2) - I_L^2, \tag{14}$$

where

$$\begin{aligned} S_{LL}^{(1)}(t_1,t_2) = &- \int_c d\tau_3 \int_c d\tau_4 \sum_{\{S_i\}} \sum_{\{\sigma_i\}} \xi_{S_2S_1}^{\sigma_1 *} \xi_{S_4S_3}^{\sigma_2 *} \xi_{S_5S_6}^{\sigma_3} \xi_{S_7S_8}^{\sigma_4} \sigma_{\sigma_4\sigma_1}(\tau_4,t_1) \sigma_{\sigma_3\sigma_2}(\tau_3,t_2) \zeta_{S_1} \\ &\times [\; g_{S_2S_5}(t_1,\tau_3) g_{S_6S_7}(\tau_3,\tau_4) g_{S_8S_3}(\tau_4,t_2) g_{S_4S_1}(t_2,t_1) - g_{S_2S_7}(t_1,\tau_4) g_{S_8S_5}(\tau_4,\tau_3) g_{S_6S_3}(\tau_3,t_2) g_{S_4S_1}(t_2,t_1) \\ &+ g_{S_4S_5}(t_2,\tau_3) g_{S_6S_7}(\tau_3,\tau_4) g_{S_8S_1}(\tau_4,t_1) g_{S_2S_3}(t_1,t_2) - g_{S_4S_7}(t_2,\tau_4) g_{S_8S_5}(\tau_4,\tau_3) g_{S_6S_1}(\tau_3,t_1) g_{S_2S_3}(t_1,t_2) \\ &+ \zeta_{S_3} g_{S_2S_7}(t_1,\tau_4) g_{S_8S_1}(\tau_4,t_1) g_{S_4S_5}(t_2,\tau_3) g_{S_6S_3}(\tau_3,t_2) - \zeta_{S_3} g_{S_2S_5}(t_1,\tau_3) g_{S_6S_1}(\tau_3,t_1) g_{S_4S_7}(t_2,\tau_4) g_{S_8S_3}(\tau_4,t_2) \\ &+ g_{S_6S_1}(\tau_3,t_1) g_{S_4S_5}(t_2,\tau_3) g_{S_8S_3}(\tau_4,t_2) g_{S_2S_7}(t_1,\tau_4) - g_{S_4S_7}(t_2,\tau_4) g_{S_8S_1}(\tau_4,t_1) g_{S_2S_5}(t_1,\tau_3) g_{S_6S_3}(\tau_3,t_2)] \end{aligned} \tag{15}$$

$$\begin{aligned} S_{LL}^{(2)}(t_1,t_2) = &- \int_c d\tau_3 \int_c d\tau_4 \sum_{\{S_i\}} \sum_{\{\sigma\}} \xi_{S_1S_2}^{\sigma_1} \xi_{S_3S_4}^{\sigma_2} \xi_{S_6S_5}^{\sigma_3 *} \xi_{S_8S_7}^{\sigma_4 *} \sigma_{\sigma_1\sigma_4}(t_1,\tau_4) \sigma_{\sigma_2\sigma_3}(t_2,\tau_4) \zeta_{S_1} \\ &\times [\; g_{S_2S_5}(t_1,\tau_3) g_{S_6S_7}(\tau_3,\tau_4) g_{S_8S_3}(\tau_4,t_2) g_{S_4S_1}(t_2,t_1) - g_{S_2S_7}(t_1,\tau_4) g_{S_8S_5}(\tau_4,\tau_3) g_{S_6S_3}(\tau_3,t_2) g_{S_4S_1}(t_2,t_1) \\ &+ g_{S_4S_5}(t_2,\tau_3) g_{S_6S_7}(\tau_3,\tau_4) g_{S_8S_1}(\tau_4,t_1) g_{S_2S_3}(t_1,t_2) - g_{S_4S_7}(t_2,\tau_4) g_{S_8S_5}(\tau_4,\tau_3) g_{S_6S_1}(\tau_3,t_1) g_{S_2S_3}(t_1,t_2) \\ &+ \zeta_{S_3} g_{S_2S_7}(t_1,\tau_4) g_{S_8S_1}(\tau_4,t_1) g_{S_4S_5}(t_2,\tau_3) g_{S_6S_3}(\tau_3,t_2) - \zeta_{S_3} g_{S_2S_5}(t_1,\tau_3) g_{S_6S_1}(\tau_3,t_1) g_{S_4S_7}(t_2,\tau_4) g_{S_8S_3}(\tau_4,t_2) \\ &+ g_{S_6S_1}(\tau_3,t_1) g_{S_4S_5}(t_2,\tau_3) g_{S_8S_3}(\tau_4,t_2) g_{S_2S_7}(t_1,\tau_4) - g_{S_4S_7}(t_2,\tau_4) g_{S_8S_1}(\tau_4,t_1) g_{S_2S_5}(t_1,\tau_3) g_{S_6S_3}(\tau_3,t_2)] \end{aligned} \tag{16}$$

$$S_{LL}^{(3)}(\tau,\tau') = i \sum_{\{S_i\}} \sum_{\{\sigma_i\}} \xi_{S_2S_1}^{\sigma_1 *} \xi_{S_3S_4}^{\sigma_2} \sigma_{\sigma_2\sigma_1}(t_2,t_1) \zeta_{S_1} g_{S_4S_1}(t_2,t_1) g_{S_2S_3}(t_1,t_2) \tag{17}$$

$$\begin{aligned} S_{LL}^{(4)}(t_1,t_2) = &- \int_c d\tau_3 \int_c d\tau_4 \sum_{\{S_i\}} \sum_{\{\sigma_i\}} \xi_{S_2S_1}^{\sigma_1 *} \xi_{S_3S_4}^{\sigma_2} \xi_{S_6S_5}^{\sigma_3 *} \xi_{S_7S_8}^{\sigma_4} \sigma_{\sigma_4\sigma_1}(\tau_4,t_1) \sigma_{\sigma_2\sigma_3}(t_2,\tau_3) \zeta_{S_1} \\ &\times [\; g_{S_2S_5}(t_1,\tau_3) g_{S_6S_7}(\tau_3,\tau_4) g_{S_8S_3}(\tau_4,t_2) g_{S_4S_1}(t_2,t_1) - g_{S_2S_7}(t_1,\tau_4) g_{S_8S_5}(\tau_4,\tau_3) g_{S_6S_3}(\tau_3,t_2) g_{S_4S_1}(t_2,t_1) \\ &+ g_{S_4S_5}(t_2,\tau_3) g_{S_6S_7}(\tau_3,\tau_4) g_{S_8S_1}(\tau_4,t_1) g_{S_2S_3}(t_1,t_2) - g_{S_4S_7}(t_2,\tau_4) g_{S_8S_5}(\tau_4,\tau_3) g_{S_6S_1}(\tau_3,t_1) g_{S_2S_3}(t_1,t_2) \\ &+ \zeta_{S_5} g_{S_2S_3}(t_1,t_2) g_{S_4S_1}(t_2,t_1) g_{S_8S_5}(\tau_4,\tau_3) g_{S_6S_7}(\tau_3,\tau_4) + \zeta_{S_3} g_{S_2S_7}(t_1,\tau_4) g_{S_8S_1}(\tau_4,t_1) g_{S_4S_5}(t_2,\tau_3) g_{S_6S_3}(\tau_4,t_2) \\ &+ g_{S_6S_1}(\tau_3,t_1) g_{S_4S_5}(t_2,\tau_3) g_{S_8S_3}(\tau_4,t_2) g_{S_2S_7}(t_1,\tau_4) - g_{S_4S_7}(t_2,\tau_4) g_{S_8S_1}(\tau_4,t_1) g_{S_2S_5}(t_1,\tau_3) g_{S_6S_3}(\tau_3,t_2)] \end{aligned} \tag{18}$$

$$S_{LL}^{(5)}(t_1,t_2) = i \sum_{\{S_i\}} \sum_{\{\sigma_i\}} \xi_{S_1S_2}^{\sigma_1} \xi_{S_4S_3}^{\sigma_2 *} \sigma_{\sigma_1\sigma_2}(t_1,t_2) \zeta_{S_1} g_{S_4S_1}(t_2,t_1) g_{S_2S_3}(t_1,t_2) \tag{19}$$

$$
\begin{aligned}
S^{(6)}_{LL}(t_1,t_2) = &-\int_c d\tau_3 \int_c d\tau_4 \sum_{\{S_i\}} \sum_{\{\sigma_i\}} \xi^{\sigma_1}_{S_1S_2} \xi^{\sigma_2 *}_{S_4S_3} \xi^{\sigma_3}_{S_5S_6} \xi^{\sigma_4 *}_{S_8S_7} \sigma_{\sigma_1\sigma_4}(t_1,\tau_4)\sigma_{\sigma_3\sigma_2}(\tau_3,t_2)\zeta_{S_1} \\
&\times \big[\; g_{S_2S_5}(t_1,\tau_3) g_{S_6S_7}(\tau_3,\tau_4) g_{S_8S_3}(\tau_4,t_2) g_{S_4S_1}(t_2,t_1) - g_{S_2S_7}(t_1,\tau_4) g_{S_8S_5}(\tau_4,\tau_3) g_{S_6S_3}(\tau_3,t_2) g_{S_4S_1}(t_2,t_1) \\
&\quad + g_{S_4S_5}(t_2,\tau_3) g_{S_6S_7}(\tau_3,\tau_4) g_{S_8S_1}(\tau_4,t_1) g_{S_2S_3}(t_1,t_2) - g_{S_4S_7}(t_2,\tau_4) g_{S_8S_5}(\tau_4,\tau_3) g_{S_6S_1}(\tau_3,t_1) g_{S_2S_3}(t_1,t_2) \\
&\quad + \zeta_{S_5} g_{S_2S_3}(t_1,t_2) g_{S_4S_1}(t_2,t_1) g_{S_8S_5}(\tau_4,\tau_3) g_{S_6S_7}(\tau_3,\tau_4) + \zeta_{S_3} g_{S_2S_7}(t_1,\tau_4) g_{S_8S_1}(\tau_4,t_1) g_{S_4S_5}(t_2,\tau_3) g_{S_6S_3}(\tau_4,t_2) \\
&\quad + g_{S_6S_1}(\tau_3,t_1) g_{S_4S_5}(t_2,\tau_3) g_{S_8S_3}(\tau_4,t_2) g_{S_2S_7}(t_1,\tau_4) - g_{S_4S_7}(t_2,\tau_4) g_{S_8S_1}(\tau_4,t_1) g_{S_2S_5}(t_1,\tau_3) g_{S_6S_3}(\tau_3,t_2)\big].
\end{aligned}
\tag{20}
$$

A set of diagrams corresponding to Eqs. (15-20) is presented in Fig. 1. Taking projections in expressions above one has to impose the normalization constraint.

Numerical results

Here we present results of several PP-NEGF calculations for non-interacting two-level systems. We utilize second order diagrammatic expansion in system-baths interaction (the non-crossing approximation), and compare the PP-NEGF results to the Hubbard NEGF simulations performed at the same (second order diagrammatic expansion) level of theory. Both approaches are tested vs. standerd NEGF which yields exact data in this case.

Fig. 2 shows example of spectral function calculation for a two-level system with one level being in the bias window, while the other completely filled (see inset). One sees that while Hubbard NEGF does provide correct result for the spectral function, PP-NEGF fails qualitatively. We stress that spectral function for the PP-NEGF is properly normalized. So, many results which depend on integral of the spectral function in energy (e.g., current) will remain relatively reliable. However, the failure is critical for energy resolved characteristics. Indeed, Fig. 3 shows that PP-NEGF is not capable to provide correct noise spectrum. Note that the failure is especially drastic at high biases. At the same time the Hubbard NEGF treated at the same level of theory (second order diagrammatic expansion in system bath coupling) appears to be quite accurate.

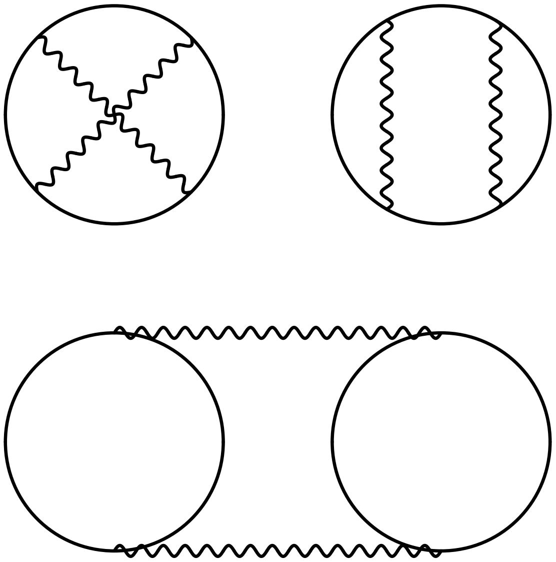

Figure 1. Diagrams for noise spectrum simulation within the PP-NEGF. Straight line indicates pseupdoparticle Green function g, wavy line stands for the standard NEGF self-energy due to coupling to contacts σ.

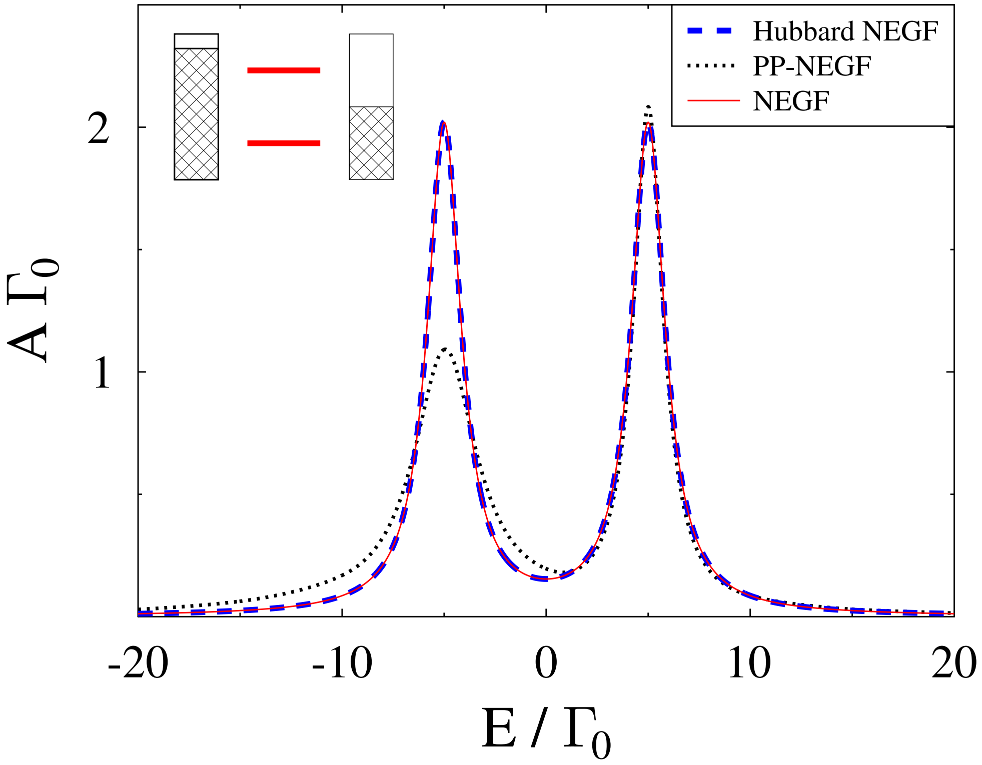

Figure 2. Spectral function for non-interacting two-level system. Hubbard NEGF (dashed line, blue) and PP-NEGF (dotted line, black) are compared with exact (NEGF - solid line, red) results.

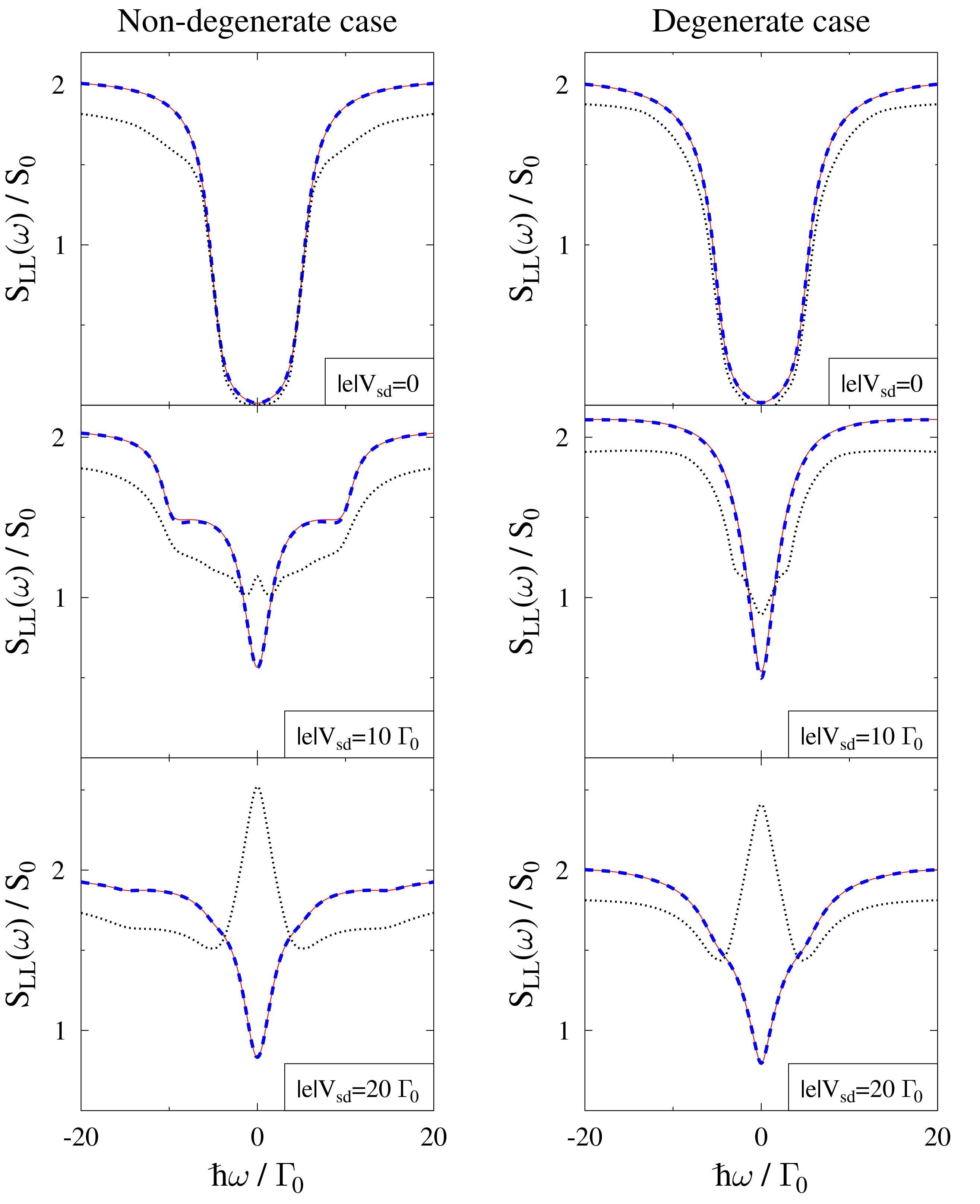

Figure 3. Noise spectra in non-degenerate (left) and degenerate (right) non-interacting two-level systems for several biases. Hubbard NEGF (dashed line, blue) and PP-NEGF (dotted line, black) simulations are compared with exact (NEGF - solid line, red) results.

References

1. Chen, F., Ochoa, M. A. & Galperin, M. Nonequilibrium diagrammatic technique for Hubbard Green functions. *J. Chem. Phys.* **146**, 092301 (2017).

2. Park, T.-H. & Galperin, M. Self-consistent full counting statistics of inelastic transport. *Phys. Rev. B* **84**, 205450 (2011).

3. Aoki, H. *et al.* Nonequilibrium dynamical mean-field theory and its applications. *Rev. Mod. Phys.* **86**, 779–837 (2014).

4. White, A. J. & Galperin, M. Inelastic transport: a pseudoparticle approach. *Phys. Chem. Chem. Phys.* **14**, 13809–13819 (2012).
